# Spatial analysis of hemorrhagic fever with renal syndrome in China

**DOI:** 10.1186/1471-2334-6-77

**Published:** 2006-04-26

**Authors:** Liqun Fang, Lei Yan, Song Liang, Sake J de Vlas, Dan Feng, Xiaona Han, Wenjuan Zhao, Bing Xu, Ling Bian, Hong Yang, Peng Gong, Jan Hendrik Richardus, Wuchun Cao

**Affiliations:** 1 Beijing Institute of Microbiology and Epidemiology, State Key Laboratory of Pathogen and Biosecurity, Beijing, China; 2 Institute of Remote Sensing Applications, Chinese Academy of Science, Beijing, China; 3 Department of Public Health, Erasmus MC, University Medical Center, Rotterdam, the Netherlands; 4 Department of Geography, University at Buffalo, USA; 5 School of Public Health, University of California, Berkeley, USA

## Abstract

**Background:**

Hemorrhagic fever with renal syndrome (HFRS) is endemic in many provinces with high incidence in mainland China, although integrated intervention measures including rodent control, environment management and vaccination have been implemented for over ten years. In this study, we conducted a geographic information system (GIS)-based spatial analysis on distribution of HFRS cases for the whole country with an objective to inform priority areas for public health planning and resource allocation.

**Methods:**

Annualized average incidence at a county level was calculated using HFRS cases reported during 1994–1998 in mainland China. GIS-based spatial analyses were conducted to detect spatial autocorrelation and clusters of HFRS incidence at the county level throughout the country.

**Results:**

Spatial distribution of HFRS cases in mainland China from 1994 to 1998 was mapped at county level in the aspects of crude incidence, excess hazard and spatial smoothed incidence. The spatial distribution of HFRS cases was nonrandom and clustered with a Moran's I = 0.5044 (*p *= 0.001). Spatial cluster analyses suggested that 26 and 39 areas were at increased risks of HFRS (*p *< 0.01) with maximum spatial cluster sizes of ≤ 20% and ≤ 10% of the total population, respectively.

**Conclusion:**

The application of GIS, together with spatial statistical techniques, provide a means to quantify explicit HFRS risks and to further identify environmental factors responsible for the increasing disease risks. We demonstrate a new perspective of integrating such spatial analysis tools into the epidemiologic study and risk assessment of HFRS.

## Background

Hemorrhagic fever with renal syndrome (HFRS) is a zoonosis caused by different species of hantavirus (HV). China is the most severe endemic country, 90% of the total HFRS cases in the world were reported [1]. Although integrated intervention measures involving rodent control, environment management, and vaccination are being implemented, HFRS remains a public health problem with 20,000–50,000 human cases annually in mainland China. The incidence of HFRS shows high variabilities at both provincial and county levels. Economic development, urbanization, human mobility, and environment and climate changes were thought to be related to incidence and spatial distribution of HFRS [21]. The HFRS incidence has been increasing in some metropolises and provincial capital cities in recent years [23]. A better understanding of the spatial distribution patterns of HFRS would help to identify areas and population at high risk.

The spatial analyses, such as spatial smoothing and cluster analysis are commonly used to characterize spatial patterns of diseases [2-9,20]. Spatial smoothing is used to reduce random variation associated with small populations and enables observations of gradients or holes of disease incidence that may not apparent from direct observation of raw data [2,10,11]. Spatial autocorrelation analysis was performed to detect significantly difference from a random spatial distribution of HFRS cases [15,18]. Spatial cluster analysis is applied to identify whether cases of disease are geographically clustered [12-14]. In this study, we conducted GIS-based spatial analyses involving spatial smoothing, exploratory spatial data analysis (ESDA) and spatial scan statistic to characterize geographic distribution pattern of HFRS cases. Spatial scan statistic was used to identify areas and population at high risk at the county level, which corrects for multiple comparisons, adjusts for the heterogeneous population densities among the different areas, detects the foci without prior specification of suspected location or size thereby overcoming pre-selection bias, and allows for adjustment of confounders [12,16,19].

## Methods

### Data collection and management

Records on HFRS cases between 1994 and 1998 were obtained from the National Notifiable Disease Surveillance System. For conducting a GIS-based analysis on the spatial distribution of HFRS, the county-level polygon map at 1:1,000,000 scale was obtained, on which the county-level point layer containing information regarding latitudes and longitudes of central points of each county was created. Demographic information based on 1995 census was integrated in terms of the administrative code [17]. All HFRS cases were geocoded and matched to the county-level layers of polygon and point by administrative code using the software ArcGIS8.3.

### GIS mapping and smoothing

To alleviate variations of incidence in small populations and areas, annualized average incidences of HFRS per 100,000 at each administrative region over the 5 year-period were calculated, and spatial rate smoothing was implemented.

Based on annualized average incidence, all counties were grouped into four categories: non-endemic area, low endemic area with annualized average incidence between 0 and 5 per 100,000, medium endemic area with the incidence between 5 and 30 per 100,000, and high endemic area with the incidence over 30 per 100,000. The four types of counties were color-coded on maps.

To assess the risk of HFRS in each county, an excess hazard map was produced. The excess hazard represents the ratio of the observed incidence at each county over the average incidence of all endemic areas, the later was calculated by the number of cases over the total number of people at risk instead of the annualized incidence of a county [18].

The technique of spatial rate smoothing was employed to annualized average incidence of HFRS. The smoothed incidence was computed from the total number of cases in a spatial "window" divided by the total number of people at risk within the "window", which was specified using a spatial weights file including both county and its neighbor counties' locations. Each smoothed incidence was calculated once the "window" core overlapped with a county center. So the first step in the analysis was to construct a spatial weights file that contained information on "neighborhood" structure of each county. The k-nearest neighbor criterion ensured each observed object had exactly the same number (k) of neighbors. In the analysis six neighbors were chosen for each county by k-nearest neighbor criterion. The second step was to load the weight file and carry out smoothing analysis [18].

To establish a continuous distribution map of HFRS, a spatial interpolation was conducted using the established county-level point layer. Inverse distance weighting (IDW) method was used due to lack of normality of distribution of annualized average incidence and difficulty of transformation (into to normal distribution).

### Spatial autocorrelation analysis

Global spatial autocorrelation analysis was performed in GeoDa0.9.5-i software. Moran's I spatial autocorrelation statistic was calculated and visualized in the form of Moran Scatter Plot. First, a contiguity-based spatial weight was constructed for each county by creating a rook contiguity weights file. Spatial autocorrelation statistics for HFRS incidence were calculated based on the assumption of constant variance. This assumption was usually violated when incidence at county level varied greatly in different populations. The Assuncao-Reis empirical bayes standardization (i.e. a function in GeoDa) was performed to adjust for the violation of the assumption. Secondly, Moran's scatter plot was produced with a spatial lag of incidence on the vertical axis and a standardized incidence on the horizontal axis. Any observation beyond two standard deviations was categorized as outlier. Thirdly, a significant test was performed through the permutation test, and a reference distribution was generated under an assumption that the incidence was randomly distributed. The number of permutation test was set to 999 and the significance level was set as 0.001.

### Spatial cluster analysis

Spatial cluster analysis was performed to detect spatial clusters of HFRS cases. "Spatial scan statistics" was used to test the null hypothesis that the relative risk (RR) of HFRS was the same between any county groups, or collection of county groups, and the remaining county groups. Areas with differing sizes were scanned without knowledge on cluster size and location to avoid selection bias. SaTScan software, designed specifically to implement this test, imposed a circular window on the map [16]. This window moved over the study region and centered on the centroid of each county. The area within the circular window varied in size from zero to some upper limit (a maximum radius of the circular window set in virtue of the proportion of the whole population) specified by the user, never including > 50% of the total population. Possible clusters were tested within the variable window around the centroid of each county group. Whenever the window finds a new case, the software calculates a likelihood function to test for elevated risk within the window in comparison with those outside the window. The likelihood function for any given window was proportional to: (d/n)d([D - d]/[D - n])(D - d) I(), where D is the total number of cases, d is the number of cases within the window, and n is the expected number of cases. If SatScan was scanning for higher incidences, the indicator function I() was 1 when cases in the window are more than expected, otherwise it would be 0. In this study, retrospective spatial cluster analysis for higher incidences was used, in which the maximum window radius was set to be smaller than 20% of the total population. Smaller maximum radius (≤ 10% of the total population) was also tried to look for possible subclusters. For each window of varying position and size, the software tested the risk of HFRS within and outside the window, with the null hypothesis of equal risk.

## Results

### Spatial distribution of HFRS in China

**Figure 1:**
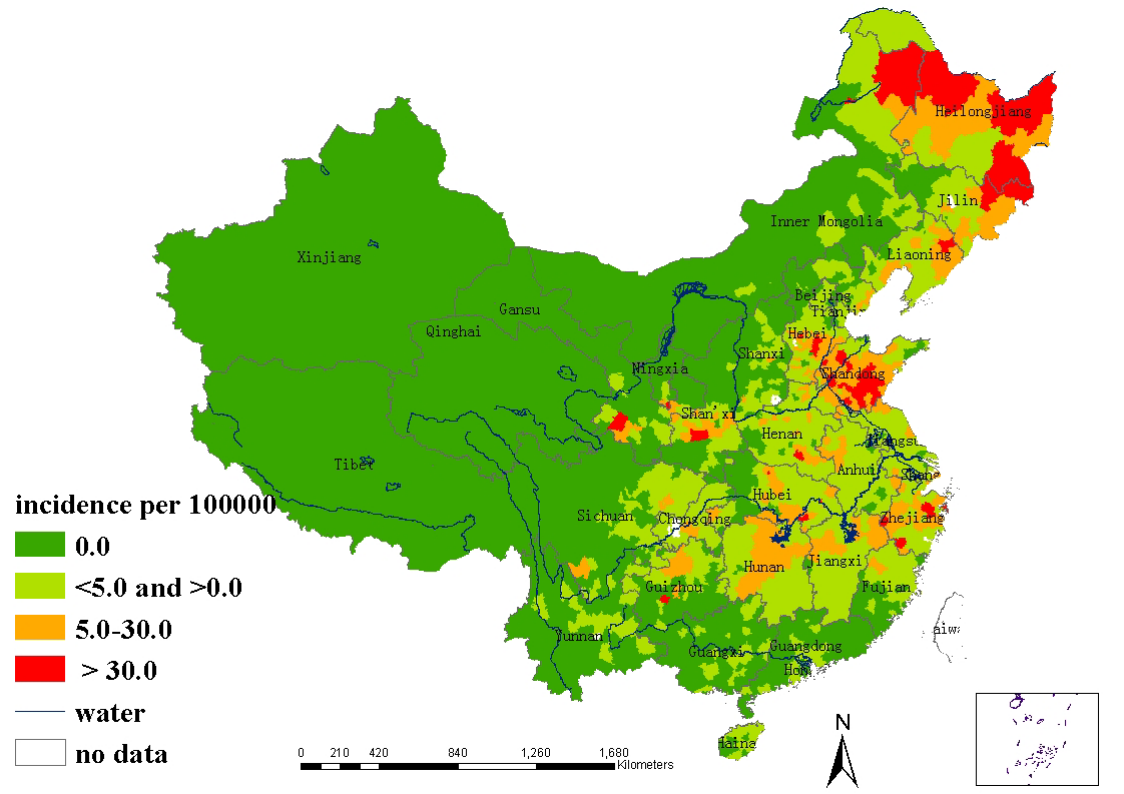
Annualized average incidence of HFRS in mainland China during 1994 – 1998.

In mainland China, a total of 257,127 HFRS cases had been reported from 1994 to 1998. Annualized average incidence at the county-level ranged from 0 to 122.117 per 100,000. Among the total 2,359 counties in China, 986 counties were non-endemic (covering 73.7% of total land and occupied by 25.1% of the total population), 969 counties were low-endemic (covering 18.8% of total land and occupied by 54.2% of the total population), 321 counties were medium-endemic (covering 5.1% of total land and occupied by 17.6% of the total population), and 83 counties were high-endemic (covering 2.4% of total land and occupied by 3.18% of the total population). The four type areas were displayed in the thematic map as showed in Figure 1.

**Figure 2:**
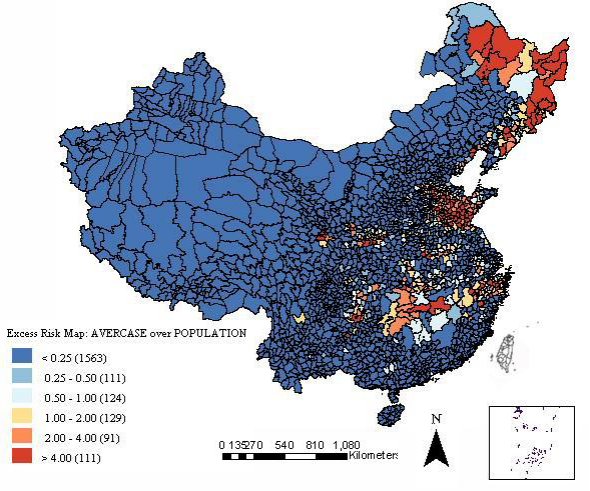
Excess hazard map of HFRS in mainland China from 1994 to 1998.

The excess hazard map showed distribution of the excess risk, which was defined as a ratio of the observed number over the expected number of cases. Counties in blue color had lower incidences than expected, as indicated by excess risk values less than 1. In contrast, counties in red color had incidences higher than expected or excess risk values greater than 1 (Figure 2). The excess risk is a non-spatial measure, which ignores the influence of spatial autocorrelation.

**Figure 3:**
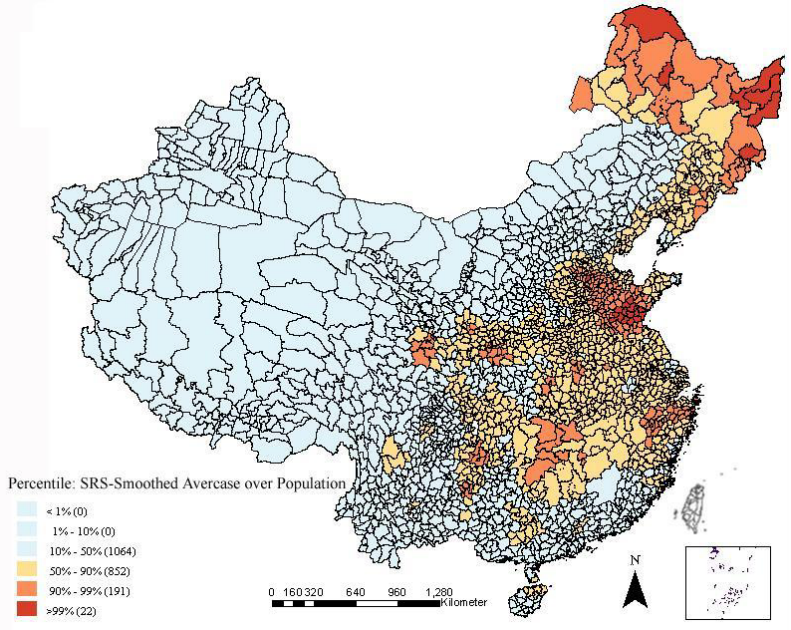
Spatially smoothed percentile map of HFRS in mainland China from 1994 to 1998.

Spatially smoothed percentile map for annualized average incidence was created for correcting the variance instability of incidences, and six neighbors identified for each county by k-nearest neighbors criterion provided the most appropriate map of smoothed incidences (Figure 3).

**Figure 4:**
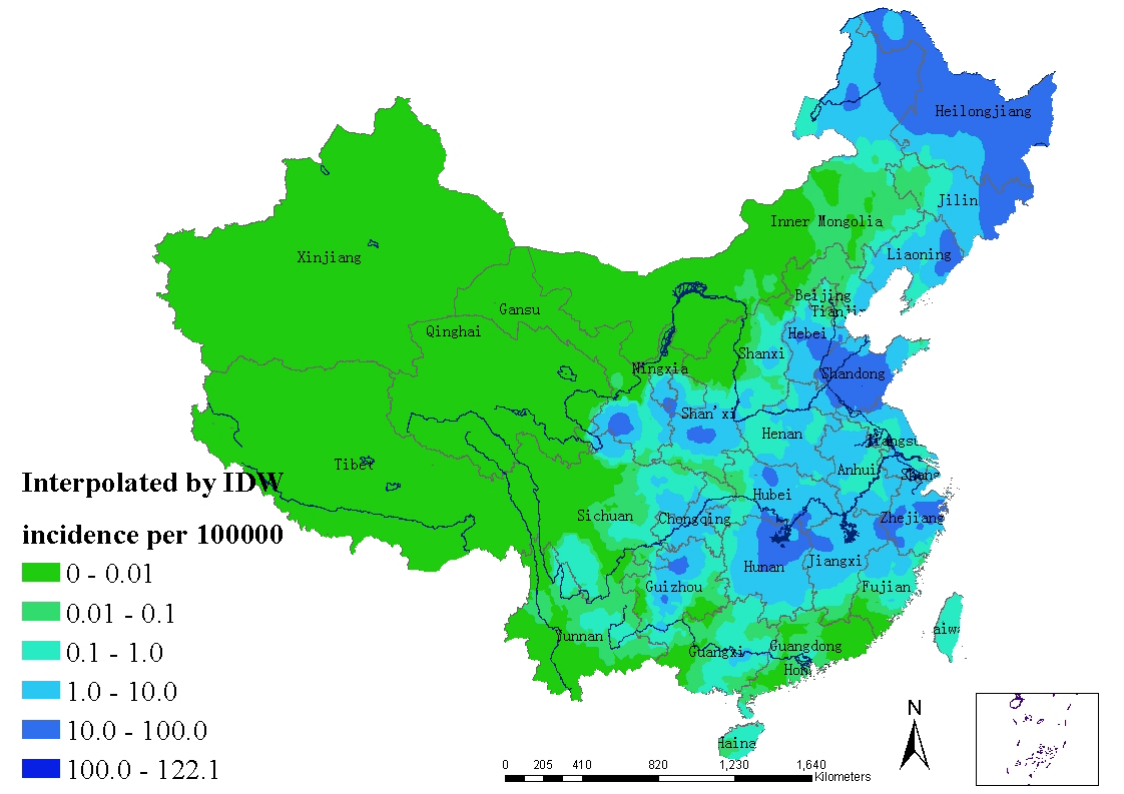
The continuous distribution map of HFRS in mainland China from 1994 to 1998.

Choropleth maps utilized administrative units (counties) with artificial boundaries to present aggregate information. Although such boundaries might have a direct impact on the reporting of disease, the county boundaries usually have nothing to do with transmission of HFRS. To deal with this problem, a continuous distribution map of HFRS was created by interpolating the centroid of each county based on its neighborhood (Figure 4). The distribution map with a small mean of prediction errors equaling -0.065 showed that the north-east, east, and centre of China were the main epidemic areas, among which high endemic areas were scattered.

### Spatial autocorrelation of HFRS in China

**Figure 5:**
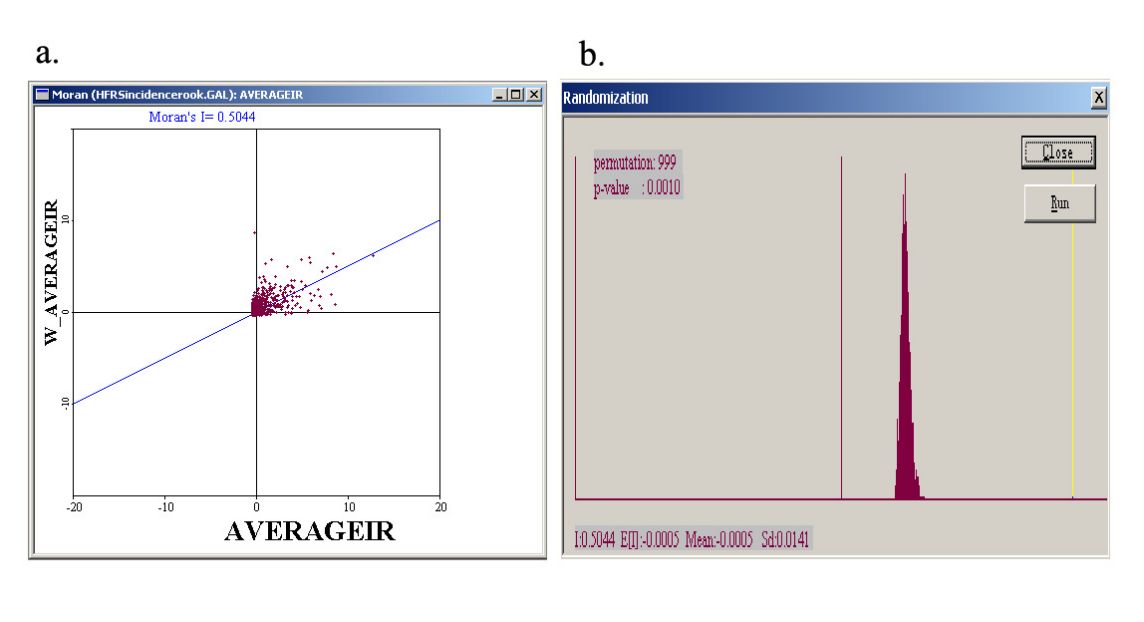
a. The Moran scatter plot for annualized average incidence of HFRS. b. The histogram for significance assessment of Moran's I.

A Moran scatter plot was created and a significance assessment through a permutation test was implemented by global spatial autocorrelation analysis for annualized average incidence of HFRS (Figure 5). The number listed on the top of the graph (0.5044) is the Moran's I statistic (Figure 5.a). A histogram was generated by performing the significance assessment of the Moran's I (Figure 5.b). In addition to the reference distribution (in brown) and the statistic (yellow bar), also listed in the graph were the number of permutations (999) and the significance level (0.001) in the upper left corner, as well as the value of the statistic (0.5044), its expected mean (E [I] = -0.0005), and the mean and standard deviation of the empirical distribution were -0.0005 and 0.0141 respectively. The statistic turned out to be significant for Moran's I at significance level of 0.001. Spatial autocorrelation analyses for annualized incidence of HFRS in mainland China from 1994 to 1998 showed that the Moran's I was significant (0.001 significance level) for each year (Table 1), implying that distribution of HFRS was spatially autocorrelated in mainland China.

**Table 1:** Spatial autocorrelation analyses for annualized incidence of HFRS in mainland China from 1994 to 1998

**year**	**Incidence(1/100,000)**	**Moran 's I**	**E [I]**	***p***
1994	5.16	0.2672	0.0005	0.001
1995	4.94	0.4374	0.0005	0.001
1996	3.65	0.5153	0.0005	0.001
1997	3.60	0.5015	0.0005	0.001
1998	3.77	0.5001	0.0005	0.001

### The distribution of HFRS clusters

**Figure 6:**
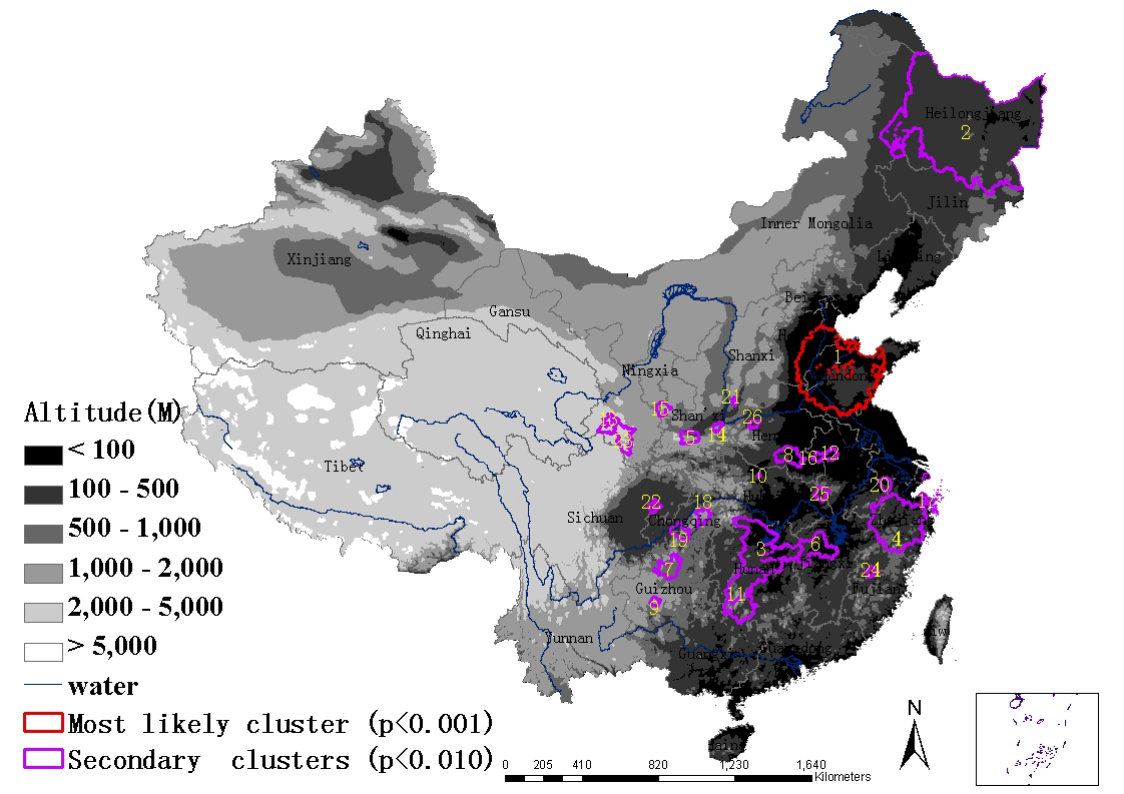
Spatial distribution of identified clusters of HFRS cases with significant higher incidences using the maximum cluster size ≤ 20% of the total population in mainland China, 1994–1998.

Using the maximum spatial cluster size of ≤ 20% of the total population, a most likely cluster and 25 secondary clusters were identified (Figure 6). The most likely cluster encompassed 127 counties in the provinces of Shandong, Hebei, and Henan, where 6.99% of the total population resides. The overall RR within the cluster was 4.776 (*p *= 0.001), with an observed number of cases of 86,424 compared with 18,094 expected cases. Twenty five secondary clusters included 196 counties with 9.52% of the total population. This excess risk within a nonrandom pattern of disease distribution was significant (*p *< 0.01).

**Figure 7:**
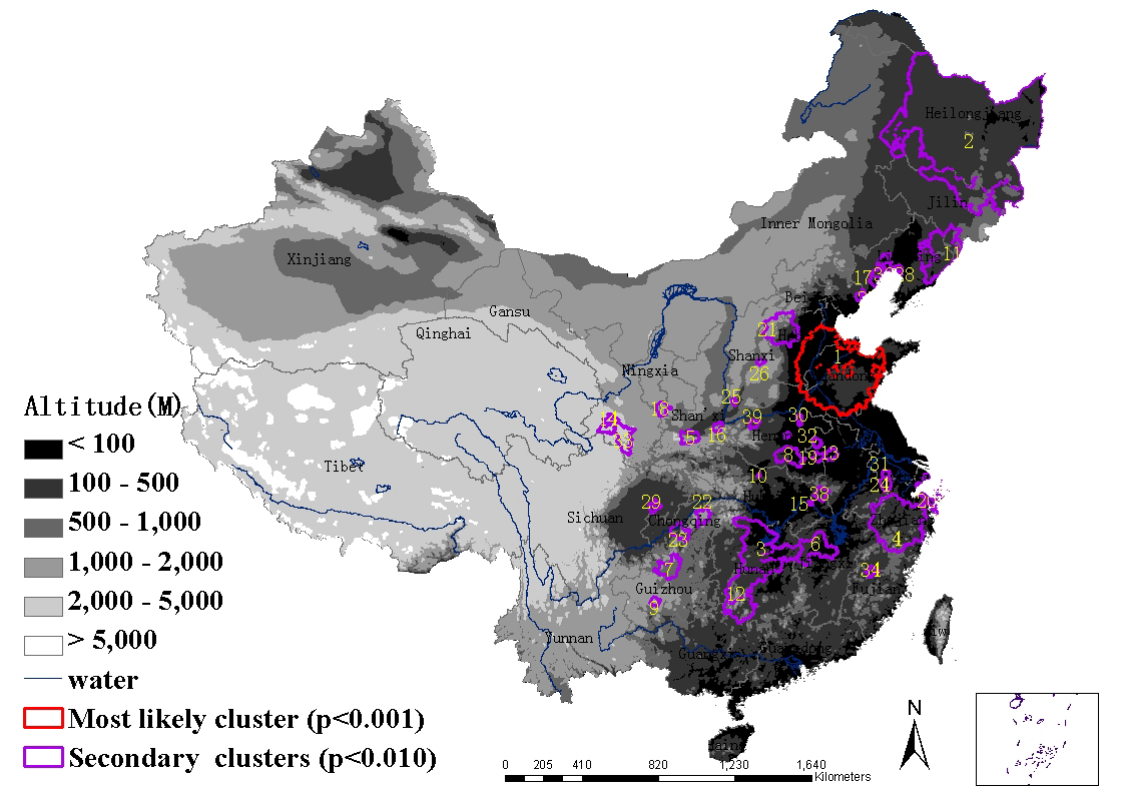
Spatial distribution of identified clusters of HFRS cases with significant higher incidences using the maximum cluster size ≤ 10% of the total population in mainland China, 1994–1998.

To investigate the possibility of smaller clusters, the same analysis was performed with a modification on maximum spatial cluster size which was defined as of ≤ 10% total population. A most likely cluster and 38 secondary clusters were identified (Figure 7). The most likely cluster was the same one as the above analysis. Thirty eight secondary subclusters included 238 counties and contained 11.42% of the total population. This excess risk within a nonrandom distribution pattern was also significant (*p *< 0.01).

## Discussion

In the study, exploratory spatial data analysis and spatial cluster analysis of HFRS were conducted at county level of mainland China. We mapped HFRS from different aspects such as crude incidence, excess risk, spatial smoothed incidence, and incidence with IDW, evaluated the spatial pattern and highlighted geographic areas with significant high incidence of HFRS in mainland China. Furthermore, this study demonstrated that additional tools necessary for disease surveillance could be provided for public health officials using existing health data, GIS and spatial scan statistics.

The study showed that the spatial distribution of HFRS in mainland China was nonrandom and clustered with a Moran's I of 0.5044 (*p *= 0.001) from 1994 through 1998. Spatial cluster analysis identified 16.51% total population and 26 areas increased HFRS risk when a maximum spatial cluster size of ≤ 20% total population was used. Additional cluster analysis based on a maximum spatial cluster size of ≤ 10% total population identified 39 subclusters occupied by 18.42% of the total population, which had statistically significant (*p *< 0.01) increased HFRS risk. The results suggest that there were "hot-spots" of HFRS in a number of areas in China, which were also the priority areas of public health planning and resource allocation for preventing HFRS. For instance, there were large areas (> 10,000 km^2^) of increased HFRS risk existed in Shandong, Hebei, Heilongjiang, Hunan, Zhejiang, Jiangxi, and Guangxi provinces, and some small areas (≤ 10,000 km^2^) with increased HFRS risk in some other provinces of central, eastern and north-eastern China.

The spatial distribution of HFRS was correlated with density, species and infection rate of rodents as the major animal reservoirs, which were influenced possibly by natural and social-economic environmental conditions such as the elevation, land use, soil type, vegetation, precipitation, atmospheric temperature, et al [21,22]. To identify and measure quantitatively the most important determinants of HFRS distribution, and to assess the burden of illness due to HFRS, more detailed epidemiological investigations need to be carried out. Clusters with significantly high incidence of HFRS identified will be helpful of investigating the underlying causes of increased risk in the identified areas, landscape attributes and identification of the environmental variables characteristic of high-risk areas with different acreage. Environmental and landscape characteristics, socio-economic factors associated with increased risk for HFRS infections need to be studied.

## Conclusion

This study has shown the presence of 'hot-spots' of HFRS in mainland China. The study has also demonstrated that using existing health data, GIS and GIS-based spatial statistical techniques could provide an opportunity to clarify and quantify the health burden from HFRS within highly endemics areas, and also lay a foundation to pursue further investigation into the environmental factors responsible for increased disease risk. To implement specific and geographically appropriate risk-reduction programs, the use of such spatial analysis tools should become an integral component in the epidemiologic description and risk assessment of HFRS.

## Competing interests

The author(s) declare that they have no competing interests.

## Authors' contributions

LF, LY, SL and SJV were involved in the conceptualization, research design, execution and write-up of the first draft of the manuscript. DF, XH and WZ contributed to database design and data analysis, BX, LB, HY, PG, JHR and WC all advised on the design of the study, and the analysis and interpretation of the results. All authors were involved in the preparation of the manuscript.

## Pre-publication history

The pre-publication history for this paper can be accessed here:


